# Impact of intense sanitization procedures on bacterial communities recovered from floor drains in pork processing plants

**DOI:** 10.3389/fmicb.2024.1379203

**Published:** 2024-05-20

**Authors:** Joseph M. Bosilevac, Manita Guragain, Darryll A. Barkhouse, Sarah E. Velez, Tatum S. Katz, Guoqing Lu, Rong Wang

**Affiliations:** ^1^U.S. Department of Agriculture, Roman L. Hruska U.S. Meat Animal Research Center, Clay Center, NE, United States; ^2^Invisible Sentinel – bioMerieux Inc., Philadelphia, PA, United States; ^3^Department of Biology, University of Nebraska Omaha, Omaha, NE, United States

**Keywords:** sanitization, pork processing, biofilm, metagenomics, *Salmonella*

## Abstract

**Background:**

Pork processing plants in the United States (US) cease operations for 24–48 h every six or twelve months to perform intense sanitization (IS) using fogging, foaming, and further antimicrobial treatments to disrupt natural biofilms that may harbor pathogens and spoilage organisms. The impact such treatments have on short-term changes in environmental microorganisms is not well understood, nor is the rate at which bacterial communities return.

**Methods:**

Swab samples were collected from floor drains to provide representative environmental microorganisms at two US pork processing plants before, during, and after an IS procedure. Samples were collected from four coolers where finished carcasses were chilled and from four locations near cutting tables. Each sample was characterized by total mesophile count (TMC), total psychrophile count (TPC), and other indicator bacteria; their biofilm-forming ability, tolerance of the formed biofilm to a quaternary ammonium compound (300 ppm, QAC), and ability to protect co-inoculated *Salmonella enterica*. In addition, bacterial community composition was determined using shotgun metagenomic sequencing.

**Results:**

IS procedures disrupted bacteria present but to different extents depending on the plant and the area of the plant. IS reduced TPC and TMC, by up to 1.5 Log_10_ CFU only to return to pre-IS levels within 2–3 days. The impact of IS on microorganisms in coolers was varied, with reductions of 2–4 Log_10_, and required 2 to 4 weeks to return to pre-IS levels. The results near fabrication lines were mixed, with little to no significant changes at one plant, while at the other, two processing lines showed 4 to 6 Log_10_ reductions. Resistance to QAC and the protection of *Salmonella* by the biofilms varied between plants and between areas of the plants as well. Community profiling of bacteria at the genus level showed that IS reduced species diversity and the disruption led to new community compositions that in some cases did not return to the pre-IS state even after 15 to 16 weeks.

**Discussion:**

The results found here reveal the impact of using IS to disrupt the presence of pathogen or spoilage microorganisms in US pork processing facilities may not have the intended effect.

## 1 Introduction

Food processing facilities harbor a wide diversity of microorganisms that persist and that may interact in multi-species biofilms, which in turn may provide an ecological niche for pathogens or spoilage organisms. Multi-species biofilms thus offer these microorganisms opportunities to colonize and gain tolerance against sanitization. Biofilm formation involving foodborne pathogens poses a serious threat to food safety and public health, while biofilms harboring spoilage organisms are a major cause of lost shelf life.

Pathogens, such as *Salmonella enterica, Listeria monocytogenes*, and Shiga toxin-producing *Escherichia coli* (STEC), develop biofilms in difficult to clean areas of food processing environments such as drains, pipes, and the back of conveyor belts (Sofos and Geornaras, 2010; Wang, 2019; Chitlapilly Dass and Wang, 2022). These surfaces attract biofilm development due to poor accessibility during sanitization steps (Mathijssen et al., 2016). Additionally, biofilms in food processing environments consist of multiple species of microorganisms, and the complex interactions within the community influences its architecture, activity, and sanitizer tolerance (Chitlapilly Dass et al., 2018; Wang, 2019). The microbial community of the biofilm forms though synergistic interactions where the microorganisms adapt to their long-term coexistence allowing a co-evolution within the niche they share (Madsen et al., 2016; Zupančič et al., 2018). Most studies related to biofilms of foodborne pathogens focus on single-species biofilms, which overlooks the fact that in the natural environment microorganisms coexist. Mixed-species biofilms are more tolerant to sanitizers than single-species biofilms or their planktonic equivalents (Wang et al., 2013; Zupančič et al., 2018; Wang, 2019).

Guidelines and best practices for IS have been described by government bodies (USDA FSIS, 2016) and industry organizations [Mikle, 2006; National Sanitation Foundation (NSF), 2024], however, the optimal approach in designing and analyzing a disinfection program requires knowledge that can only be supplied by processors. When and how best to perform an IS must take into account activities in each area of the environment, the type of products present, and the management of antimicrobial sanitization (Doyle et al., 2017; Maillard and Centeleghe, 2023). Processing plants in the United States (US) periodically perform intense sanitization (IS) to disrupt biofilms that may harbor pathogens and spoilage organisms. This procedure typically involves stopping operations at the plant for 24 to 48 h while cleaning, foaming, and fogging of the plant environment with two component sanitizers takes place. Due to the necessary interruption of plant activities and cost, an IS is only performed every 6 or 12 months, while in between IS, nightly cleaning and sanitization using more moderate sanitizers takes place.

The impact of IS on the short-term changes in environmental microorganisms is not well understood. When the microbial contamination in a Chinese pork abattoir environment was characterized before and after disinfection, abundant microbial diversity was observed with notable changes in composition following the disinfection (Sui et al., 2023). Disinfection effectively reduced microbial diversity and abundance in the processing plant, but bacteria were not eradicated. Some bacteria survived the disinfection processes while others exhibited an increase in abundance (Sui et al., 2023). The succession of bacterial communities following IS has not been described, although when the environment of a newly opened pork plant was monitored for one and a half years, an increase in total abundance and alpha diversity was detected after the first 2 months (Cobo-Díaz et al., 2021). The study here was designed to examine the initial impact of IS on the microorganisms recovered from pork processing plant drains and follow their succession over three months following the IS. The recovered microorganisms were characterized for their biofilm-forming ability, resistance to sanitization, ability to protect pathogens if present, and community profile.

## 2 Materials and methods

### 2.1 Sample collection and processing

Floor drain samples were collected at two US pork processing plants (designated Plant H and Plant M) that were scheduled to perform intense sanitization (IS). In these two plants, IS involved ceasing operations for 48-h where first the harvest, then processing sides of the plant were cleaned and sanitized. The IS used foam and spray applications of Decon7 (D7; Coppell, TX) according to the guidelines of the manufacturer. Briefly, after a general cleaning of all plant surfaces (equipment, floors, walls, etc.) that targeted all visible matter, Decon7 was applied to the plant environment that was at approximately 20°C. DECON7 is a multi-part disinfectant, where Part One is described as a blend of water soluble surfactants in water: alkyl-dimethyl-benzyl ammonium chloride (3.2% wt/vol), pentamethyl-N-alkyl trimethylene diammonium chloride (1% wt/vol), diethylene glycol monobutyl ether (1.6% wt/vol), 1-Dodecanol (0.8% wt/vol), isobutanol (1.0% wt/vol), Propylene Glycol (20% wt/vol), potassium hydroxide (3.2% wt/vol), and potassium bicarbonate (10% wt/vol); it is activated by the addition of an equal volume of Part Two: hydrogen peroxide (7.98% wt/vol), and 1/50 volume of Part Three the food grade solvent diacetin (>99%), in a 49:49:2 final ratio. Following exposure to the cleaner (approximately 8 h), all surfaces were rinsed with hot (80°C) water. Finally, an antimicrobial rinse (400 ppm peroxyacetic acid, pH 3.5) was used throughout the areas to complete the IS. Each evening, the plants performed overnight cleaning and decontamination that consisted of washing all surfaces, rinsing with hot (80°C) water and a quaternary ammonium compound (QAC; 300 ppm).

Samples were collected 4 to 6 h after activities (harvest, cooler emptying of carcasses, processing/fabrication) had begun and used cellulose sponges (Speci-sponge; Nasco, Atkinson WI), each wetted with 10 mL of buffered peptone water (BPW; Becton Dickinson, Sparks MD). All drains were standard industrial drains located at low points of the floor for the collection and removal of run off. Drains in separate cooler bays (4 at each plant) and in the processing room along different product lines: ham (2 at each plant), loin, and butt were selected. Appropriate drains were identified as those that were safely accessible and located in an area where visible run off was collected (i.e., not in a corner or under moving equipment). Drains were assigned an arbitrary type A-E (Supplementary Figure S5). Then, the covering grate was removed and the drain visually inspected for biofilm (a viscous, shiny film). The underside of the grate and interior surfaces were sampled to collect bacteria and biofilms if observed from an area of ~500 cm^2^ by vigorously swabbing with the sponge, turning it over halfway through the process. At the time of collection, the temperature of each sampling area was recorded with an infrared thermometer (Nubee NUB8550AT, Cole-Parmer, Vernon Hills, IL).

Samples were collected 2–3 days prior to IS at both plants, then at Plant M, all drains were sampled on the day of IS, and 3 days and 1, 2, 4, 8, 12, and 16 weeks post-IS. At Plant H on the day of IS, two coolers and one processing drain were sampled. Distance and travel time to Plant H resulted in a modified schedule of post-IS sample collection, where all drains were resampled 3 days, and 1, 3, 7, 11, and 15 weeks post-IS. Due to issues of safety, maintenance, and accessibility, some cooler drains at Plant H were not sampled repeatedly, but the next nearest drain was selected as a substitute when required. Sponges were sealed in their Whirl-Pak bag then transported to the laboratory on wet ice in a cooler. Upon receipt, each drain sample was thoroughly hand massaged and portions removed for bacterial enumeration, biofilm assays, and DNA extraction as described below. Unused portions were mixed with sterile glycerol (17% final concentration) and frozen. In total, 131 samples were collected, 72 at Plant M and 59 at Plant H. At plant M, each of four coolers and four processing locations (two ham lines, a loin line, and a butt line) were sampled pre-IS, immediately after IS, and during seven follow-up visits (see above). At plant H, similar locations were sampled (four coolers, two ham lines, a loin line, and a butt line); however, only coolers 3 and 4, and ham line 1 were sampled immediately after IS. Therefore, there were eight samples collected from these locations (due to altered follow-up visit schedule) and seven from all others (cooler 1, cooler 2, ham line 2, butt line, and loin line).

### 2.2 Enumeration of bacterial groups

Each sample was serially diluted to determine total mesophile count (TMC), and psychrophilic bacteria (PSY), as well as *Enterobacteriaceae* (EB), coliforms (CF) and *E. coli* (EC) using Petrifilm AC (3 M, St Paul, MN) for TMC and PSY; Petrifilm EB for EB; and Petrifilm ECC for CF and EC. Petrifilm were incubated 24 h at 37°C for EB, CF and EC; 48 h at 30°C for TMC and 10 days at 7°C for PSY as described previously (Chitlapilly Dass, 2020). All values were log transformed and presented as Log_10_ CFU/100 cm^2^ in Supplementary Figures S1, S2.

### 2.3 Screening of biofilm formation

The biofilm-forming potential of each sample was screened using a crystal violet (CV) assay. Ten-fold dilutions of primary drain samples in Lenox broth (LB, Acumedia Manufactures Inc., San Bernardino, CA) without salt (LB-NS) medium were transferred to a 96-well plate (200 μL per well). Samples were incubated at temperatures relative to those recorded during collection. Plant H Coolers 3 and 4 were incubated at 2°C for 10 days, Plant H Coolers 1 and 2 and Plant M Coolers 1–4 were incubated at 7°C for 7 days; all processing samples (Ham, Butt, and Loin lines) at Plant M were incubated at 12°C for 5 days, while processing samples from Plant H were incubated at 15°C for 5 days. Then, supernatant was removed, and the biofilm was gently washed two times with sterile phosphate buffered saline (PBS, Sigma Aldrich, St. Louis, MO). Microtiter plates were dried in oven at 65°C for 30 min, followed by incubation with 50% crystal violet (CV; Millipore Sigma, Burlington, MA) for 20 min. Plates were washed to remove excess CV then allowed to dry at room temperature for 1 h. Biofilm associated CV was extracted with 200 μL of 85% ethanol and quantified by measuring absorbance at 570 nm. Five replicates were set up for each drain sample, averaged, and presented as arbitrary biofilm strength units in Supplementary Figures S3, S4.

### 2.4 Sanitizer treatment of drain biofilm samples

**Figure 1:**
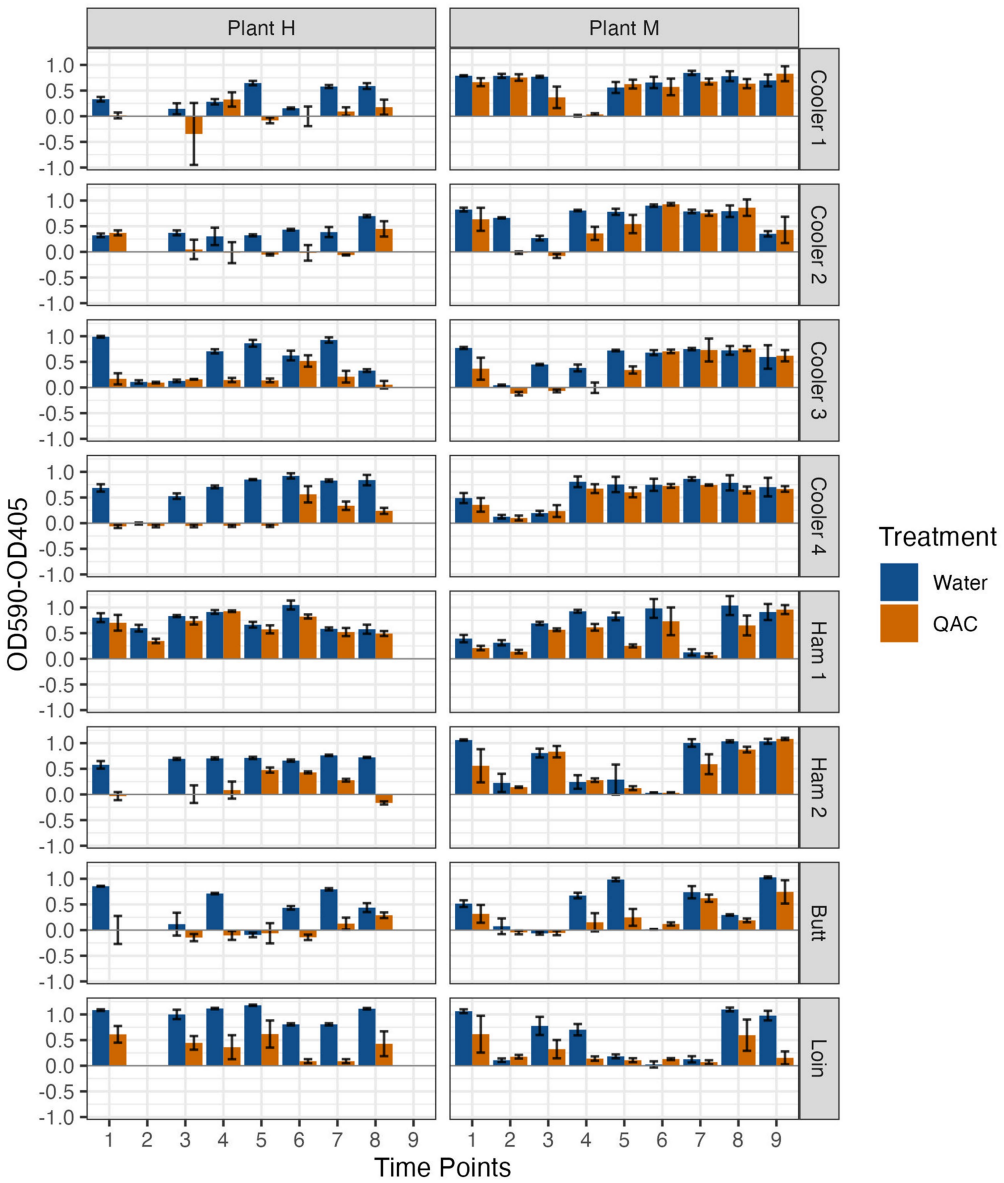
Resistance to 300 ppm quaternary ammonium compound (QAC) compared with a water control of biofilms formed by microorganisms recovered from drains located at Pork Processing Plants H and M before and after intense sanitization procedures. Time points 1–8 (Plant H) and 1–9 (Plant M) on X axis; 1 = 2 to 3 days pre-intense sanitization (IS); 2 = day of IS; 3 = 2 to 3 days post-IS; 4–8 = 1, 3, 7, 11 and 15 weeks post-IS, respectively, for plant H and 4–9 = 1, 2, 4, 8, 12 and 16 weeks post-IS, respectively, for Plant M. *No day of IS samples were collected from Coolers 1 and 2 at Plant H, and only Ham Line 1 Day of IS sample was collected at Plant H.

Individual drain samples were enriched as described previously (Chitlapilly Dass, 2020) with modifications. Briefly, primary drain samples were grown in LB-NS medium, with orbital shaking at 200 rpm, for 5–10 days at temperatures as described above. When samples became visibly turbid (OD600 ~ 1.0), glycerol stocks of the enrichments were prepared and stored at −20°C until use. For sanitizer treatment experiments, an enriched glycerol stock was thawed, diluted 1:100 in LB-NS, and transferred to 10 wells of a 96-well plate followed by incubation at their respective temperatures and times. At the end of the incubation period, supernatant was removed, and biofilm was gently washed with sterile PBS. The biofilms in five of the wells were exposed to freshly prepared QAC (Vanquish, Total Solutions, Milwaukee WI) at a final concentration of 300 ppm for 1 min, followed by two washings with PBS. For non-treated control, QAC was substituted with sterile water in the other five wells. To assess the viability of biofilm after QAC treatment, 200 μL of Dey-Engley broth (DE; BBL, Difco, Sparks, MD) was added to each well and incubated at 25°C for 14 h. At the end of the incubation period, optical densities of samples were read at 405 nm (purple color of DE broth) and 590 nm (yellow color due to growth). Growth was calculated as OD590-OD405 (Wang et al., 2017), and the mean and standard deviation were determined and shown as arbitrary units in Figure 1.

### 2.5 Pathogen protection in biofilm

Protection of *Salmonella enterica* by biofilms was evaluated using the sanitizer treatment method described above with some modifications. *S. enterica* isolates EL262—1 (serovar Enteritidis), CJ1—8B (serovar Infantis), and CJ3—7B (serovar Typhimurium), which had been previously recovered from pork products or Plant H, were individually grown overnight in Tryptic soy broth (TSB, BBL, Difco) at 37°C. A pool of these strains was prepared by mixing equal volumes of each *S. enterica* culture. Then, the pool was added to each thawed drain enrichment (1:100) and biofilms were developed in four wells of a 96-well plate, two treated with QAC and two treated with sterile water as described above. Then, the levels of *S. enterica* protected by the biofilms were determined using a GENE-UP^®^ *Salmonella* SLM2 kit (bioMérieux, St. Louis MO) following the manufacturer’s recommended protocol for presence absence testing. Briefly, after the final two washings, each well was resuspended in 30 μL PBS. Template was prepared by transferring 20 μL of this suspension to GENE-UP^®^ Lysis Kit (bioMérieux), followed by incubation at 95°C for 5 min, shaking at 2500 rpm. 10 μL of the lysate was used as a template. Averages of the Cp values reported by the GENE-UP^®^ system at the completion of the SLM2 assay were used to determine the percent protection of the *S. enterica* from QAC. This was done by converting the differences of Cp of QAC treatment and its water control to Log_2_ fold change, then calculating the percentage of fold change. No change in Cp between QAC and water is equal to 100% protection of *S. enterica* by growth in the biofilm, 1 Cp difference equal to 50% protection, 2 Cp difference equal to 25% protection, and so on. Percentage protection ≥75% by a biofilm was considered highly protective, ≤25% non-protective, and values between 26 and 74% considered moderately protective.

### 2.6 Metagenomic shotgun sequencing and analysis

**Figure 2:**
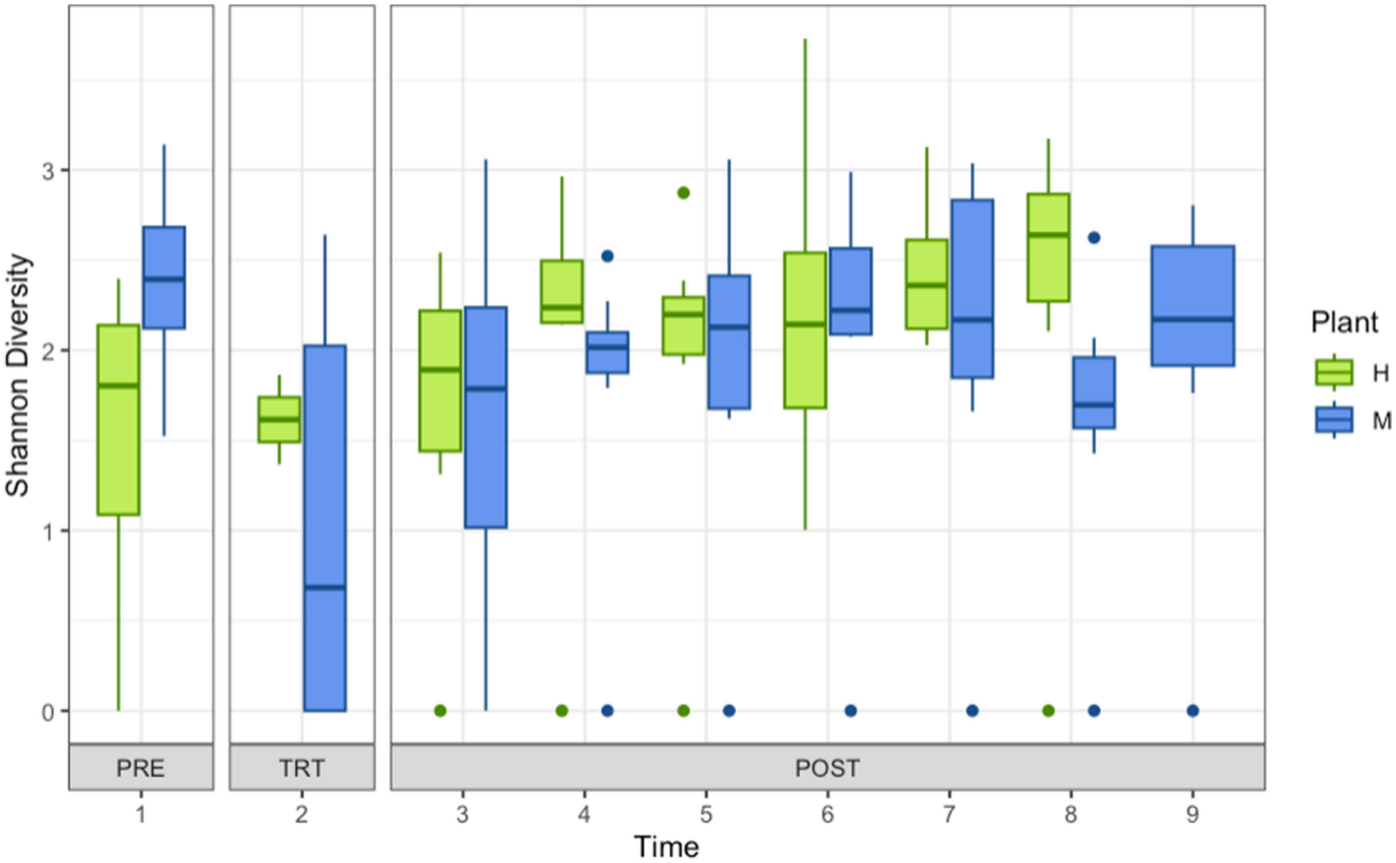
Shannon diversity of shotgun metagenomic sequencing of organisms recovered from drains located in coolers and carcass fabrication rooms at Pork Processing Plants H and M before and after intense sanitization (IS) procedures. Time points 1–9 on x-axis; 1 = 2 to 3 days pre-intense sanitization (PRE); 2 = day of IS (TRT); 3 = 2 to 3 days post-IS (POST); 4–8 = 1, 3, 7, 11, and 15 weeks post-IS Plant H; 4–9 = 1, 2, 4, 8, 12, and 16 weeks post-IS Plant M.

DNA was extracted for metagenomic sequencing as described previously (Palanisamy et al., 2023) using Qiagen Powerlyzer Powersoil extraction kits and including a bead beating step (1,200 rpm, 3 min) in a MP Biomedicals (Solon, OH) FastPrep96 (with modified aluminum holder) to ensure optimum lysis of organisms present. Purified DNA was quantified using a Qubit 4 Fluorometer (Thermo Fisher Scientific, Waltham, MA) then submitted for shotgun metagenomic sequencing (Invisible Sentinel, Philadelphia PA). DNA from each sample (100 ng) was prepped for sequencing using an Illumina DNA Prep Kit (96 Samples) and NexteraTM DNA Indexes (96 Samples) (Illumina, San Diego, CA) according to the instructions of the manufacturer. Prepped DNA was analyzed for fragment size distribution using an Agilent BIoAnalyzer 2,100 and an Agilent High Sensitivity DNA Kit (Agilent Technologies, Santa Clara, CA), and concentration was measured using the Qubit 4 Fluorometer. The prepared DNA samples were pooled, denatured, diluted to appropriate concentration, and loaded into the MiSeq instrument (Illumina, San Diego, CA) using an Illumina MiSeq Reagent Kit v3 (600 cycle) per manufacturer’s instructions. The paired-end sequencing run was set to 2×250 cycles. The FASTQ files generated were used for all downstream analyses using CosmosID (Germantown MD) pipeline and bacteria identified filtered to the genus level. Relative abundance based on abundance score (equal to number of reads) was tabulated using Microsoft Excel to generate relative abundance plots (Figure 2) and organism tables (Supplementary Table S1).

### 2.7 Statistical analysis

Crystal violet biofilm formation (OD570 nm) and QAC tolerance DE broth growth (OD405-590) were analyzed for mean and standard deviation using Microsoft Excel (version 16.79.2, Microsoft Corp. Redmond WA), plotted and visualized. The biofilm formation and QAC tolerance were compared using a one-way analysis of variance (ANOVA) with a post-Dunnett’s multiple comparisons test using R Studio integrated development environment for R (R Core Team, 2022; RStudio Team, 2022). *p*-values less than 0.05 were considered statistically significant.

To gain a greater understanding of what in the plant environment may predict differences in biofilm communities, we performed three sets of analyses on the metagenomic data. The metagenomic analyses and data visualizations were performed in the R Studio using the packages tidyr (Wickham et al., 2023), vegan (Oksanen et al., 2022), stats and base (R Core Team, 2022), and factoextra (Kassambara and Mundt, 2020). The first analysis was to determine whether the plant (H or M), time point of collection, treatment (IS), site sample collected, temperature, drain type, or location (cooler vs. processing) predict the species richness of the biofilm, we calculated the number of species per biofilm (function vegan::specnumber, Oksanen et al., 2022). We then fit a series of linear models using each of the above variables as predictors of species richness. Specifically, a t-test was run to determine whether species richness differed significantly by plant (functions stats::var.test and stats::t.test, R Core Team, 2022); one-way analyses of variance (ANOVA) were run to determine whether species richness differed significantly by time, treatment, site, drain type, or location (function stats::aov, R Core Team, 2022); and a linear model was fit to determine whether temperature is a significant predictor of species richness (function lm::stats, R Core Team, 2022).

To determine whether these variables predict the Shannon diversity (Hill, 1973) of the biofilm, the Shannon diversity of each was calculated (function vegan::diversity, Oksanen et al., 2022). We then proceeded by developing linear models as above using each of the variables to predict differences among Shannon diversity indices. To determine whether any of these variables predict different community compositions of the biofilms, we ran a series of permutational multivariate analyses of variance [PERMANOVA (Anderson, 2017), function vegan::adonis2 (Oksanen et al., 2022)] using each of the variables of interest as a predictor for community composition. PERMANOVA tests for differences between community compositions based on a dissimilarity measurement, in our case the Bray-Curtis index (Ricotta and Podani, 2017). It can be intuitively understood as a test in which each species within the biofilm is represented as a single axis or dimension, and the centroid of each community is the point in multidimensional space that describes which species are in the community. The distances between the centroids of each group of communities are then compared, with the null hypothesis that the centroids are equal for all groups (Anderson, 2017). Here, the groups are defined by the variables of interest (plant, time, treatment, site, temperature, drain type, and location). Core biomes for each group were calculated as a function of both occurrence and abundance (Neu et al., 2021). Any organism which had greater than 1% abundance across at least 50% of samples was classified as a member of the core biome (Lahti et al., 2017).

## 3 Results and discussion

### 3.1 Intense sanitation impacts on indicator organisms

The initial measure of IS impact was determined through single point in time measurements of TMC, PSY, EB, CF, and EC (Supplementary Figures S1, S2). In coolers, PSY was the most abundant group of organisms measured, and in some cases closely matched by the TMC. IS performed at Plant H had little to no effect on the numbers of organisms recovered from cooler drains with only Cooler 3 demonstrating a reduction of 1–1.5 Log_10_ CFU. However, IS was observed to impact numbers of EB, *CF*, and EC more so with reductions of 2 to 3 Log_10_ CFU. In coolers at Plant M, IS introduced significant reductions of PSY and TMC ranging from 2 to 5 Log_10_ CFU. In some cases, this was matched with a corresponding decrease in EB, CF, and EC. Organism counts in Cooler 1 at Plant M were further decreased 2 days after IS. This is likely due to the fact that this cooler had not gone back into use by the time the post-IS samples were collected, whereas Coolers 2, 3, and 4 at Plant M were full of carcasses when samples were collected at this point.

The impact of IS on organisms measured in drains located in fabrication was similar to those observed for coolers except TMC was more abundant in many cases than PSY. This was likely due to temperature differences. Temperatures recorded at cooler sample collection points at the two plants averaged 5 to 5.5°C, whereas those recorded in processing areas averaged 17°C at Plant H and 12°C at Plant M. PSY are not as enriched in the warmer fabrication rooms as compared to the coolers. Indoor microbial environments are influenced by occupants and their activities as well as the building layout and operations taking place (Adams et al., 2016), and at Plant M organisms in drains located near the ham processing lines were not as impacted by IS as drains near the butt and loin processing lines. Organisms recovered from drains near ham lines were reduced by 1 to 2 Log_10_ CFU, whereas reductions were 4 Log_10_ CFU near the Butt Line and greater than 6 Log_10_ CFU near the Loin Line. A result due to the level of traffic around ham lines compared with the others. Drains near ham lines at Plant M had considerable forklift and loader traffic, while the drains near the Butt Line and Loin Line were located between processing tables and only had light worker foot traffic.

Based solely on these observational indicator counts, IS, in some situations, can impact the levels of organisms present and in some cases have an impact lasting 16 weeks or more. Most studies that examine contamination or the impact of IS/disinfection in pork processing facilities do not make note of indicator organisms (Campos Calero et al., 2020; Zwirzitz et al., 2020; Cobo-Díaz et al., 2021; Sui et al., 2023). In areas such as hospitals, replacing plumbing to disrupt recurring outbreak of carbapenem-resistant *Enterobacteriaceae* (CRE) led to a decline in CRE in patients; however, the environment was found to be rapidly recolonized by CRE and patient infections with CRE returned (Decraene et al., 2018). The observations in many of the drains examined here agree with this model, while other sample sites disagreed with different indicator organism groups emerging. Further follow-up and monitoring would be needed to learn more about these differences. However, organism counts alone do not reflect the phenotype of the biofilm the recovered organisms may produce; therefore, all drain samples were assessed for their ability to form a biofilm.

### 3.2 Biofilm formation by organisms recovered from drains

The biofilm-forming ability and the strength of biofilm formed by organisms recovered at Plants H and M showed significant differences (*p* < 0.05) in biofilm strength between sites within the plant (Supplementary Figures S3, S4). At Plant M, all sites showed similar results except Cooler 4, which formed significantly weaker biofilm than all other sites. At Plant H, biofilms formed by organisms recovered from Cooler 1 and Cooler 2, were stronger (*p* < 0.05) than biofilms formed by all other sites, while biofilms formed by organisms from the Loin Line and Butt Line were the weakest (*p* < 0.05). Biofilms formed by organisms from Ham Line 1 were stronger (*p* < 0.05) than those formed from Ham Line 2, but neither were different (*p* > 0.05) from biofilms formed by organisms from Coolers 3 and 4.

The IS procedure was seen to significantly disrupt biofilm formation in Coolers 3 and 4 at Plant H (Supplementary Figure S3), but the organisms collected 3 days post-IS were as strong of biofilm formers as the pre-IS organisms. At 1 week post-IS, Coolers 3 and 4 at Plant H showed a weaker biofilm-forming ability, and this may have correlated to the reductions in indicator counts. The patterns of results of biofilm formation by organisms recovered from cooler drains at Plant M were different in each of the cooler bays. In all cases except Cooler 4 at Plant M, the pre-IS organisms were strong biofilm formers. Organisms collected at this location were weak biofilm formers before and after the IS, remaining so until week 4 when the population shifted to one forming strong biofilm.

Organisms recovered from fabrication drains also presented differing biofilm formation. Despite having remarkable differences in organism counts in samples collected from the fabrication room at Plant M, the biofilm-forming ability of the recovered organisms was little changed following the IS (Supplementary Figure S4). However, at Plant H, IS resulted in various outcomes at each location. IS slightly weakened biofilm-forming ability by Ham Line 1 organisms that returned to strong biofilm-forming ability 3 days post-IS. The biofilm-forming ability of organisms from Ham Line 2 was intermediate and remained so throughout the 15 weeks of follow-up. Biofilms formed by organisms recovered near the Butt Line were significantly weakened in their ability to form biofilms post-IS, while Loin Line organisms were initially weak biofilm formers, but post-IS became strong biofilm formers that weakened over time. IS inducing strong biofilm formation has been described at a beef processing plant where the post-IS organisms were found to be strong biofilm formers (Wang et al., 2024). It was hypothesized that although the total number of organisms was reduced by IS those able to tolerate IS were stronger biofilm formers and then able to expand with less competition and reoccupy the drain environment (Wang et al., 2024). The results collected at Plants H and M show that the impact of IS on organisms can be variable and that other factors are likely involved.

### 3.3 Sanitizer tolerance and pathogen protection of biofilms following IS

Given the varied organism levels and biofilm-forming strength of the recovered organisms pre- and post-IS among all the samples, all samples were taken forward to determine their resistance to QAC treatment (Figure 1). Variable results between plants and between locations within the same plant were again observed. At Plant H, the Cooler 2, Ham Line 1, and Loin Line initial pre-IS biofilms were tolerant to QAC, while at Plant M all pre-IS biofilms demonstrated some or full tolerance to QAC. The IS only markedly disrupted the QAC tolerance of Plant H Cooler 2 and Plant M Coolers 2, 3, and Butt Line biofilms with all others similar to the water wash control. While the biofilm QAC tolerance rapidly returned in one or two weeks to Plant M Coolers 2 and 3, tolerance did not return to the Plant M Butt Line or Plant H Cooler 2 biofilms until six and seven weeks after the IS.

The biofilm QAC tolerance measured may have been a transient response not related to the IS treatment. For instance, tolerance of QAC was decreased in biofilms formed by samples collected at Ham line 2 (15 weeks post-IS) and Loin Line (5 and 7 weeks post-IS) of Plant H, and Ham Line 1 (2 weeks post-IS) at Plant M. Furthermore, short lived (single collection time point) increases in QAC tolerance were also observed in biofilms formed by samples collected from Cooler 1 (3 weeks post-IS) and Cooler 3 (5 weeks post-IS) at Plant H. Indeed, QAC tolerance of biofilms formed by samples from Ham Line 2 at Plant H was observed to increase (weeks 3 and 7 post-IS) then decrease again, while Loin Line biofilms at Plant M varied by time point. These variations may have been more related to the community of bacteria present as well as those being introduced rather than a response to the IS. Differing combinations of bacterial species have been shown to resist disinfection (Kocot and Olszewska, 2020; Pang et al., 2020); and bacterial communities in a pork processing plant were described to be influenced by the incoming carcasses as well as changes in the hygienic practices of the processing plant (Campos Calero et al., 2020; Sui et al., 2023). Zwirzitz et al. (2020) tracked the likely source of bacteria in a pork processing plant to the carcasses. Such transient changes in the bacteria passing through Plants H and M as well as differing plant activities over time may be responsible for these unanticipated observations.

In addition to measuring the biofilm sensitivity to QAC, these sets of samples were inoculated with *Salmonella* to determine to what extent the biofilm protected it from QAC (Table 1). We considered *Salmonella* protection of ≥75% to be strong protection and ≤ 25% to be non-protective, with the range of 26–74% being moderate protection. Samples from Plant M were more protective of *Salmonella* than those collected at Plant H. The *Salmonella* protection did not have any clear relationship to biofilm strength, or QAC tolerance. In fact, samples that showed greater QAC sensitivity appeared to offer increased *Salmonella* protection. This warrants further analysis and examination in light of the community profiles.

**Table 1:** Protection of *Salmonella* by biofilms formed by organisms recovered from drains located in coolers and carcass fabrication rooms.

	Time point								
Plant H	1	2	3	4	5	6	7	8	9
Cooler 1	67	nd	0	51	30	43	82	22	
Cooler 2	72	nd	42	100	74	80	39	49	
Cooler 3	74	61	87	49	42	45	83	53	
Cooler 4	61	56	57	91	67	83	55	79	
Ham line 1	1	0	83	10	8	15	8	1	
Ham line 2	41	nd	53	74	95	42	81	59	
Butt line	100	nd	100	54	2	100	63	44	
Loin line	54	nd	81	54	11	16	21	49	
Plant M									
Cooler 1	100	100	100	98	74	35	70	19	24
Cooler 2	100	33	89	54	100	38	74	78	7
Cooler 3	53	100	76	100	100	75	0	86	62
Cooler 4	77	81	55	66	70	62	37	67	76
Ham line 1	54	73	77	98	100	12	34	82	25
Ham line 2	76	82	62	97	94	20	56	77	10
Butt line	18	78	70	66	80	7	34	66	31
Loin line	99	59	94	92	91	19	78	48	9

Values represent percent protection from quaternary ammonium compound (QAC; 300 ppm) based on log2 fold change as compared to water control as measured by quantitative PCR for the invA gene. Heat map shows strong *Salmonella* protection (≥75%; red), non-protective (≤ 25%; green), and moderate protection (26–74%; yellow). Time points 1–8 (Plant H) and 1–9 (Plant M) are as follows: 1: 2 to 3 days pre-intense sanitization (IS); 2: day of IS; 3: 2 to 3 days post-IS; 4–8: 1, 3, 7, 11, and 15 weeks post-IS, respectively, for Plant H while 4–9: 1, 2, 4, 8, 12, and 16 weeks post-IS, respectively, for Plant M. nd: no data; at Plant H only Cooler 3, Cooler 4, and Ham Line 1 IS samples were collected.

### 3.4 Community shifts following is measured by shallow shotgun metagenomics

The impact of changes in biofilm phenotypes such as strength of biofilm, sensitivity to QAC, and protection of pathogens has been shown to be a result of the community structure (McBain et al., 2003; Raghupathi et al., 2018; Chitlapilly Dass et al., 2020). A diversity of members has been shown to stabilize environmental microbial communities and prevent invasion by certain species of pathogens (Kennedy et al., 2002; Blaser and Falkow, 2009; Blaser, 2016). Mahnert et al. (2019) showed cleaning and disinfecting resulted in a loss of microbial diversity and a population that shifted from Gram-positive to Gram-negative bacteria. Although IS had variable impacts on the number of bacteria recovered from the various drains in our study (Supplementary Figures S1, S2), the progression of recolonization started with different organisms and in some cases a community shift of different organisms in the following weeks. To further elucidate these changes, we performed shallow shotgun metagenomics on all samples to determine the relative abundances of genera in each.

The number of genera present post-IS was reduced but Shannon diversity was not different (Figure 2). Lower relative abundance and community complexity suggest effectiveness of disinfection, but as noted by others, targeted disinfection measures may be insufficient (Sui et al., 2023). Analysis showed that time point, sample site, temperature, or location (cooler vs. processing) did not significantly predict a difference in how many genera were in the biofilm, nor predict a difference in Shannon diversity of the biofilms. Only the Drain type (Supplementary Figure S5), plant (H or M), and treatment (Supplementary Figure S6) produced significantly different community compositions. The contribution of the community structure to biofilm strength, QAC sensitivity, and pathogen protection is a complex system of multi-species interactions (Wang, 2019). Previous studies suggested that disrupting or altering a preexisting environmental microbial community may lead to unintended consequences because less tolerant species are eliminated leaving only the more tolerant and space for colonization by invasive groups (Kotay et al., 2020). Furthermore, the phenomena in microbial ecology of pulse dynamics and disturbances (Jentsch and White, 2019; Burton et al., 2020) may also explain the shifts in biofilm composition as well as its phenotype. Further analysis and modeling are required to determine whether particular genera appear to have positive or negative influences on the biofilm formation and biofilm phenotypes. The inclusion of more sample sites too would aid in analysis. The samples examined here were limited to coolers and processing areas. The inclusion of samples from harvest and storage areas may provide additional data that reveals further interesting and useful information on the impacts of IS on processing plant communities.

**Figure 3:**
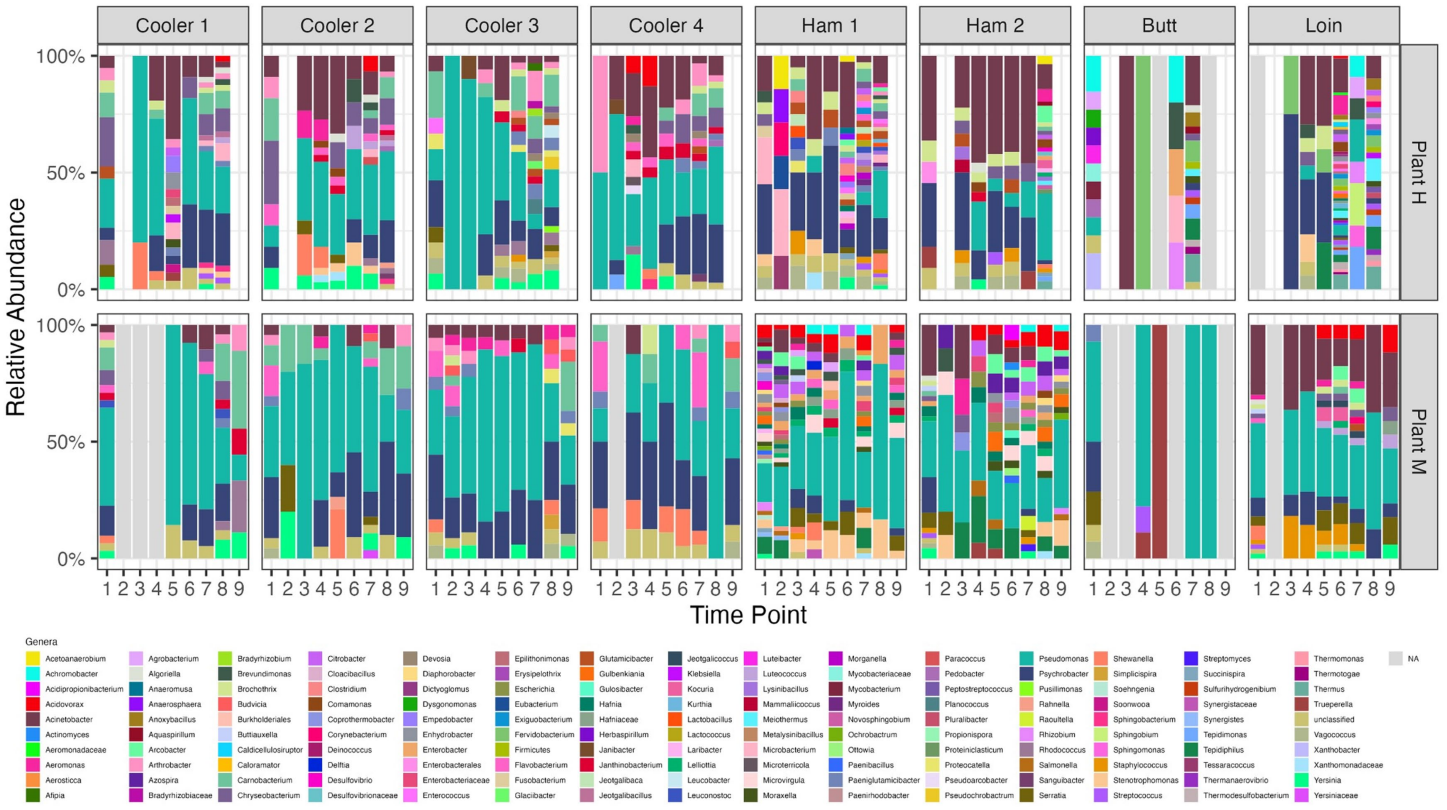
Relative abundance of genera identified by shotgun metagenomic sequencing of organisms recovered from drain samples at pork processing Plant H and M before and after intense sanitization procedures. Time points on x-axis; 1–8 (Plant H) and 1–9 (Plant M); 1 = 2 to 3 days pre-intense sanitization (IS); 2 = day of IS; 3 = 2 to 3 days post-IS; 4–8 = 1, 3, 7, 11, and 15 weeks post-IS, respectively, for plant H and 4–9 = 1, 2, 4, 8, 12, and 16 weeks post-IS, respectively, for Plant M. Open blue framed bars “N/A” are samples that failed to yield sequencing results due to low DNA recovery from organisms present. *No day of IS samples were collected from Coolers 1 and 2 at Plant H and only Ham Line 1 Day of IS sample was collected at Plant H.

The predominant genera (Figure 3) found in coolers at both plants were *Psychrobacter*, *Acinetobacter, Arthrobacter*, and *Pseudomonas*, one of the most prevalent taxa present in food processing facilities (Xu et al., 2023). Plant H coolers had more *Chryseobacterium* and *Brochothrix*, while Plant M coolers had more *Flavobacterium*, and *Carnobacterium*. At certain time points, low abundances of Yersinia were detected. Coolers at Plant M had fewer genera present compared with coolers at Plant H. Opposite to what was found for communities recovered from coolers, the fabrication floor drains at Plant H had more genera present than Plant M. IS reduced the number present in fabrication drain samples with the number increasing over time following IS. *Psychrobacter*, *Pseudomonas*, and *Acinetobacter* were again the most abundant genera found in fabrication samples; however, there were differences between the two plants and between fabrication sample sites (ham vs. butt vs. loin). Similarly, *Pseudomonas* spp. were the most frequently found and abundant among both meat and dairy products and their environments. However, levels of the same species differed by processing and type of sample, which suggested to the authors that strains of the same species were able to adapt differently to their changing environment (Stellato et al., 2017).

We observed *Pseudomonas* being one of the initial groups to reestablish itself following IS. *Pseudomonas* has a strong tolerance to harsh growth conditions such as low temperatures and high stress (Langsrud et al., 2016). This was reported to allow them to survive and outcompete companion organisms in areas of fabrication and storage holding areas where the temperature was low (Zhao et al., 2004; Belk et al., 2022). Examining microbial succession of a newly opened pork cutting plant showed the microbiome on different surfaces becoming more specific to that surface. A microbiome dominated by a limited number of environmental bacteria formed a core microbiome that was mostly shared among samples from different rooms and surfaces, but which evolved over time (Cobo-Díaz et al., 2021). Considering the core microbiomes of Plants H and M, *Pseudomonas* and *Psychrobacter* were members of both, with *Acinetobacter* also part of the Plant H core microbiome. Sui et al. (2023) also identified *Psychrobacter* and *Acinetobacter* as the dominant flora before and after disinfection in a pig slaughterhouse.

The core microbiomes in coolers and processing areas differed (Supplementary Table S2). Core microbiomes of coolers included *Pseudomonas, Psychrobacter, Carnobacterium*, and *Arthrobacter* at both plants, with Plant H coolers also having *Acinetobacter* and *Yersinia* as part of its core microbiome. Processing areas had much narrower core microbiomes, with that of Plant H composed of *Psychrobacter* and *Acinetobacter*, and Plant M only *Pseudomonas*. Furthermore, individual locations had core biomes that differed from one another. For instance, core microbiomes of coolers 2 and 3 at Plant H could be distinguished by the presence or absence of *Yersinia*, *Chryseobacterium*, *Carnobacterium*, and *Vagococcus*. Ham lines at Plant M both had *Pseudomonas* as part of their core microbiome, but Ham line 1 included *Stenotrophomonas*, *Enterobacter*, *Citrobacter*, and *Acidovorax* while Ham line 2 include *Tepidiphilus* and *Azospira* instead. The Butt Line samples though in our study had non-informative reads at some time points and transient colonization at others so no identifiable core microbiome was identified.

Meat plants harbor a wide range of environmental microorganisms and the variations between drain sites within a plant are likely due to the operations and processing taking place in the nearby areas. It was shown that drain microbial communities in different areas of a meat processing facility could be differentiated from one another by the function of a room, the local activity taking place, temperature, and the sources of incoming microbes (Belk et al., 2022; Palanisamy et al., 2023). We noted significant differences in communities between plants and the drain types in use. The structure of the various drain types allows liquids to flow differently (Supplementary Figure S5). The drains with the side trap and clean-out accessory (Type C) hold much more liquid than those that drain directly into the plumbing system of the processor. The standing liquids in Type C drains may supply unique local environmental conditions and shelter communities from stresses such as those occurring with an IS protocol, thus allowing certain organisms to thrive and dominate a local community compared with the other drain types. The marked differences in Coolers and Processing areas are supported by a report that found abundance profiles of spoilage organisms and potential pathogens did not agree between processing and packaged pork products. Also noted was that daily hygiene practices did not impact the main phyla colonizing the plant environment (Hultman et al., 2015).

Shallow shotgun sequencing as we performed here provided data to identify the metagenomic community and identify predominant organisms in each sample. Since shotgun sequencing sequences all the DNA available in a sample, additional information can be found, such as the identities of virulence factors of pathogens present or antimicrobial resistance and stress tolerance genes that persisting organism may possess. Thus shallow shotgun sequencing provides a picture of the genetic phenotype of the communities. Our focus, however, was to assess the impact of IS on the community members and how that changed over time. In some IS and post-IS samples, community structure at the genera level was not resolved due to the lack of informative reads for taxonomic group assignments. This may be attributed to the shotgun metagenomic approach, which requires a high-depth sequencing.

The results reflect that the IS procedure may not be efficient in reducing the risk associated with the formation of biofilms, nor prevent pathogen recolonization. Alternate forms of biofilm control are under investigation in or laboratory. Indeed, specific IS procedures may be necessary for coolers compared with the processing area of the plants, and each plant may require its own customized approach to IS and biofilm control (Mikle, 2006; National Sanitation Foundation (NSF), 2024). The metagenomic analyses of these plants are ongoing with additional samples from storage and harvest areas. It is hypothesized that this will lead to an increased understanding of each environment and what approaches of IS may be best suited. It should be noted though that not all biofilms are unwelcome. Some biofilm communities that form in meat processing facilities have been found to exclude the colonization of pathogens (Chitlapilly Dass et al., 2020; Wang et al., 2024). Therefore, IS procedures may be useful to disrupt an unwelcome biofilm, allowing a more desirable biofilm community to be seeded in its place.

## 4 Conclusion

We assessed the impact of IS on bacteria recovered from floor drains located on the coolers and fabrication rooms at two pork processing plants (H and M). IS procedures at both plants disrupted bacteria present, but to different extents depending on the plant and the area studied. The impact of IS on organisms in coolers was varied, with reductions of 2 to 4 Log_10_ CFU, and required 2 to 4 weeks to return to pre-IS levels. The results observed near fabrication lines were mixed, with little to no significant changes at Plant H, while at Plant M, the Butt Line and Loin Line had 4 to 6 Log_10_ CFU reductions. The Log_10_ CFU reductions following IS often, but not always, correlated with the biofilm-forming strength. Tolerance to 300 ppm quaternary ammonium compound (QAC) by the biofilms formed by the recovered bacteria varied between plants and among areas of the plants as well as with the variations possibly due to the composition of the microbial community. Future studies will focus on isolating and characterizing individual members of the community and assessing their contributions to the community phenotype. Community profiling showed that IS led to new community compositions that in some cases did not return to the pre-IS state even after 15 to 16 weeks. The results here suggest that the IS procedure is not efficient in reducing the risk associated with the formation of biofilms or in preventing pathogens. Thus, alternate biofilm control strategies should be evaluated in these types of facilities. However, the desire to reduce biofilm formation and activity should be tempered, as there are beneficial biofilms that fill processing niches and prevent the establishment of problem organisms. Thus, the communities that did not support *Salmonella* need further analyses to determine what a desired community resembles, and experiments are needed to determine if such a supporting biofilm can be purposely seeded into post-IS conditions.

## Data availability statement

The authors declare that the data supporting the findings of this study are presented within the article. The fastq.qz files of the Illumina paired-end sequencing and metadata are originated from meat processing plants, and authors are obliged to maintain confidentiality, preventing the public deposition of these sequences. However, in case of a reasonable request, the authors are fully capable and willing to make these data available through file sharing. The data was generated under a non-disclosure agreement with meat processors, and we can share data with those willing to be bound by the same nondisclosure agreement.

## Author contributions

JB: Conceptualization, Funding acquisition, Investigation, Methodology, Project administration, Resources, Supervision, Visualization, Writing – original draft, Writing – review & editing. MG: Investigation, Methodology, Visualization, Writing – review & editing. DB: Data curation, Methodology, Resources, Writing – review & editing. SV: Data curation, Formal analysis, Methodology, Writing – review & editing. TK: Data curation, Formal analysis, Software, Visualization, Writing – review & editing. GL: Formal analysis, Writing – review & editing. RW: Investigation, Methodology, Resources, Supervision, Writing – review & editing.
