# Supplementary material for: Impact of intense sanitization procedures on bacterial communities recovered from floor drains in pork processing plants
Source: Front Microbiol. 2024 May 20;15:1379203. doi: 10.3389/fmicb.2024.1379203 (PMC11144920; doi:10.3389/fmicb.2024.1379203)

Supplemental Figure 4. Biofilm forming strength of microorganisms recovered from drains located in the carcass fabrication rooms at Pork Processing Plants H and M before and after intense sanitization procedures.

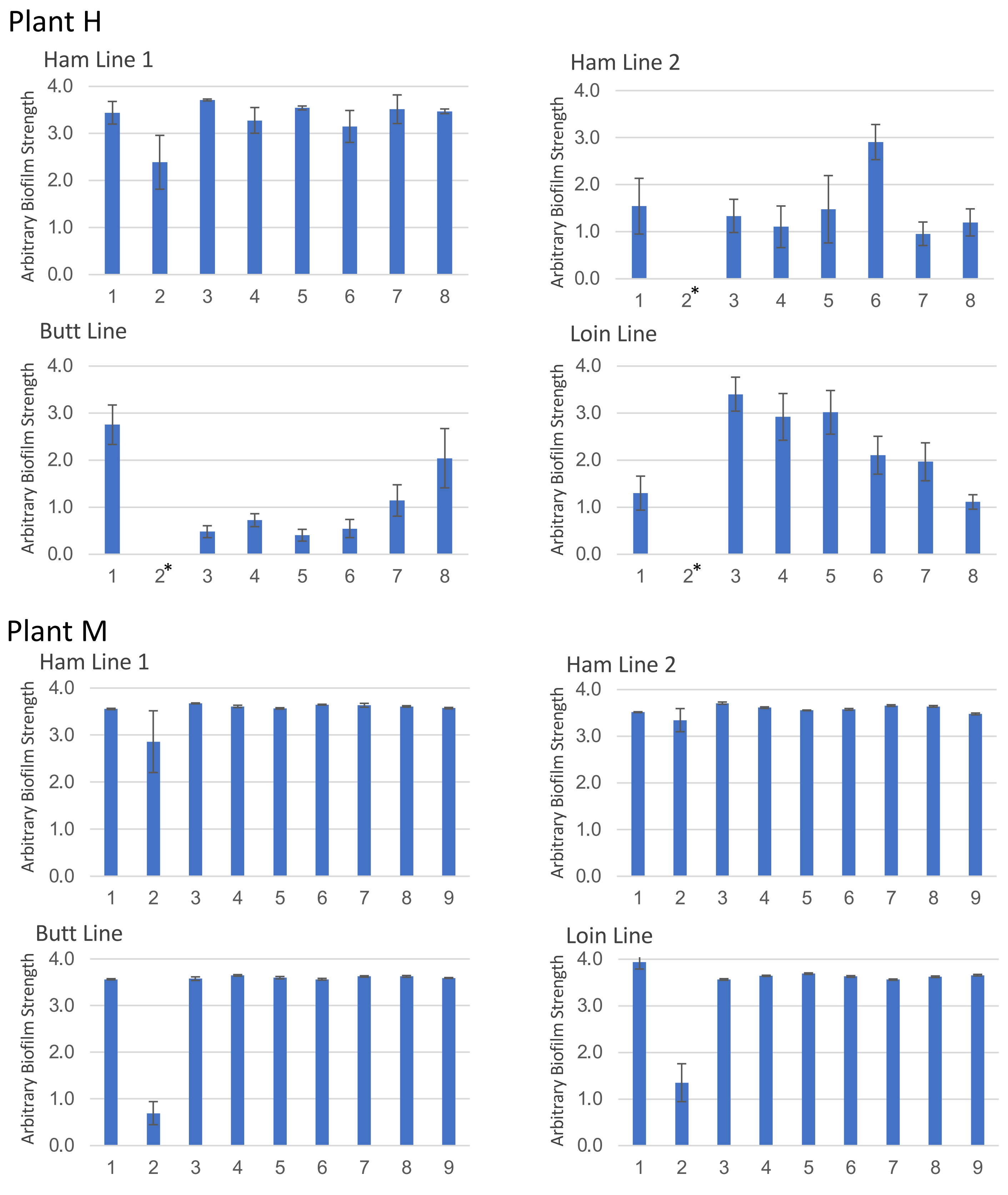

Supplement: Supplementary file 6 [file Data_Sheet_4.pdf]
