# Supplementary material for: Impact of intense sanitization procedures on bacterial communities recovered from floor drains in pork processing plants
Source: Front Microbiol. 2024 May 20;15:1379203. doi: 10.3389/fmicb.2024.1379203 (PMC11144920; doi:10.3389/fmicb.2024.1379203)
Supplement: Supplementary file 7 [file Data_Sheet_5.pdf]

Supplemental Figure 5. Drain Types (A-D) and community composition

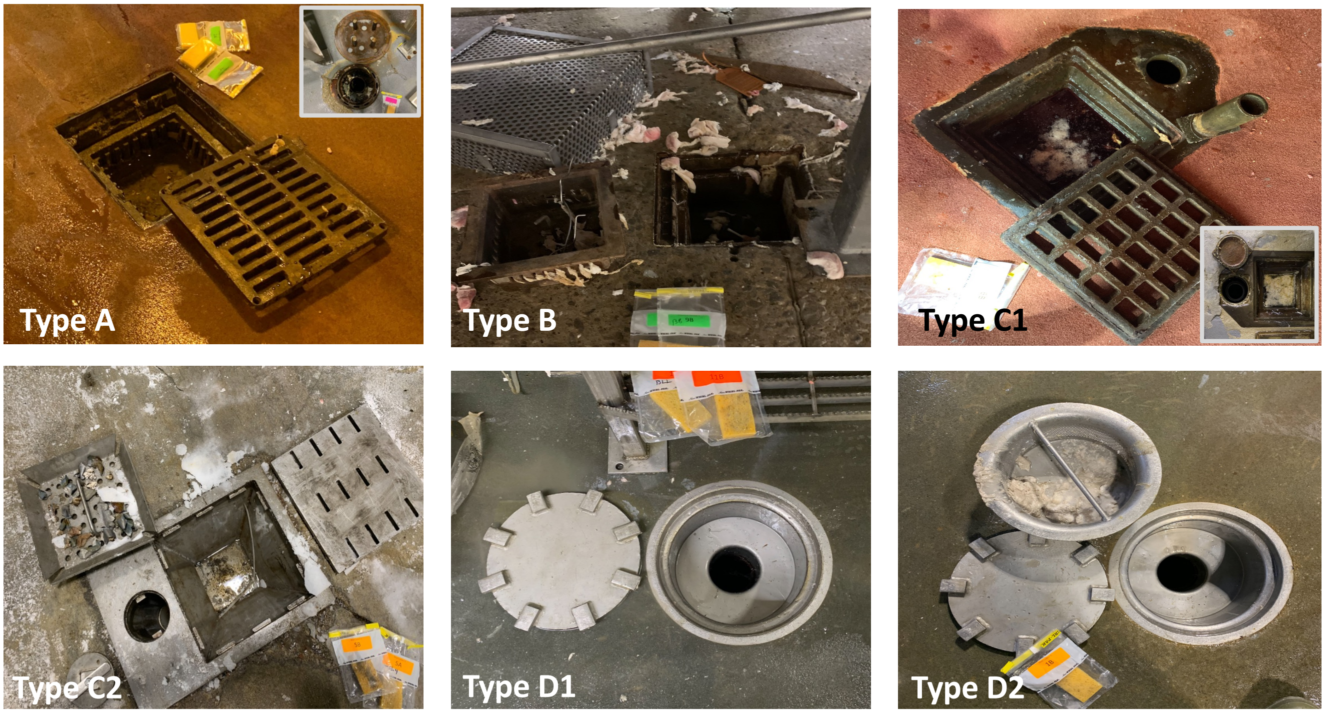

Type A, steel floor drain, with removable grate and no basket. Most often square, but occasionally round (inset). Type B, Square steel floor drain, similar to Type A but with removable basket to catch debris. Type C1, Square steel floor drain, side trap draining, with removable grate, fixed basket and side clean out. Clean out plugs/covers can vary (unset). Type C2, similar to Type C1, but with removable basket to catch debris, grate coverings may vary. Type D1, round stainless-steel floor drain with raised cover. Type D2, Round stainless-steel floor drain with raised cover and removable basket.

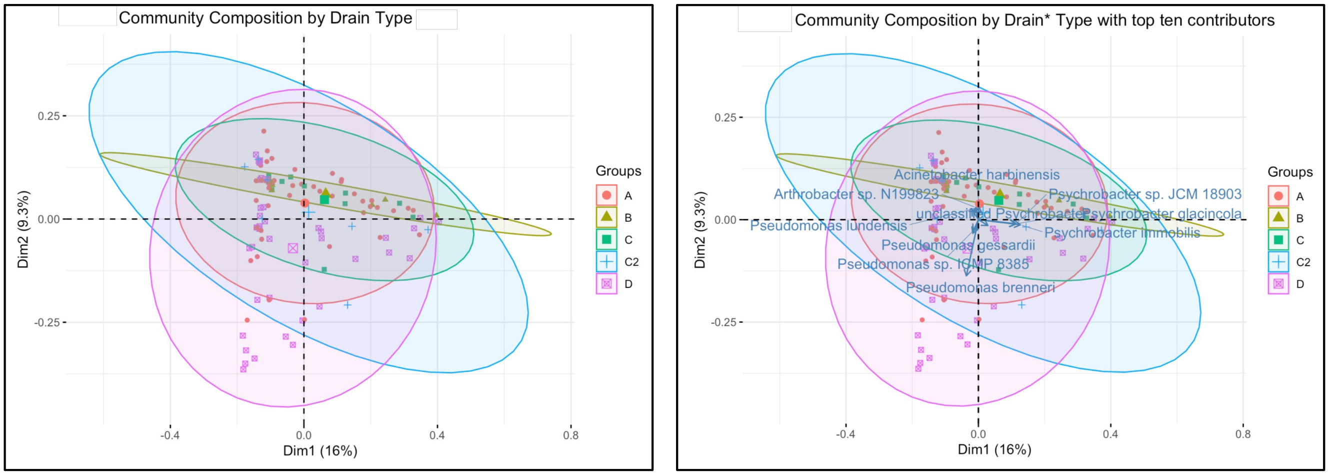

Community composition of drain types A-D plotted with and without top ten contributing organisms. Drain types show significantly different community compositions.
