# Supplementary material for: Impact of intense sanitization procedures on bacterial communities recovered from floor drains in pork processing plants
Source: Front Microbiol. 2024 May 20;15:1379203. doi: 10.3389/fmicb.2024.1379203 (PMC11144920; doi:10.3389/fmicb.2024.1379203)
Supplement: Supplementary file 8 [file Data_Sheet_6.pdf]

Supplemental Figure 6. Community Composition

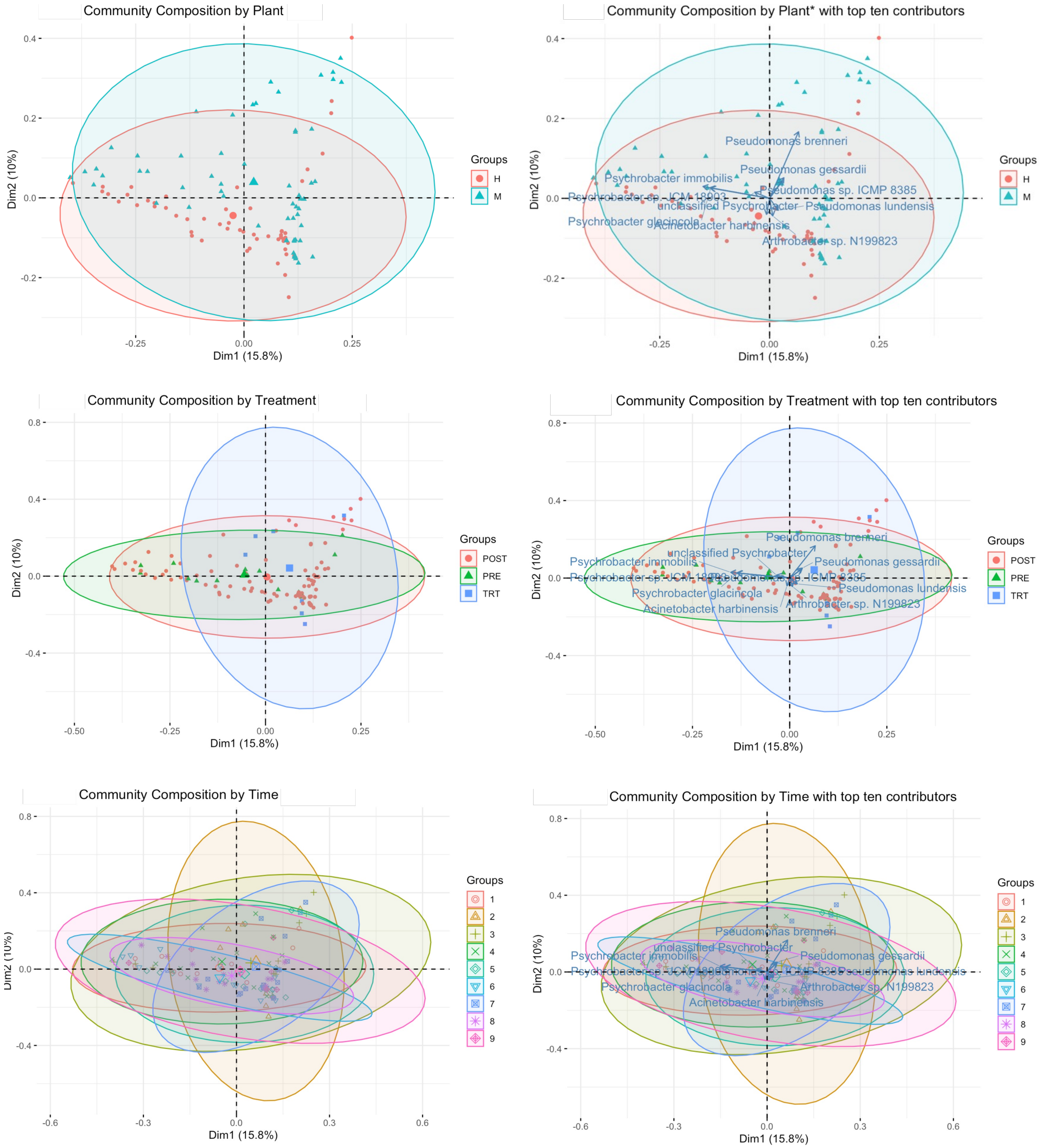

Community composition based on shotgun metagenomic sequencing of organisms recovered from drains located in coolers and carcass fabrication rooms at at pork processing Plants H and M before and after intense sanitization (IS) procedures. Plotted with and without top ten contributing organisms by Plant (H or M), treatment (PRE, TRT, POST) and time points 1-8 for Plant H and 1-9 for Plant M. Analysis by plant and treatment were significantly different*.
